# The Language of Creativity: Validating Linguistic Analysis to Assess Creative Scientists and Artists

**DOI:** 10.3389/fpsyg.2021.724083

**Published:** 2021-11-19

**Authors:** Sana Tariq Ahmed, Gregory J. Feist

**Affiliations:** Department of Psychology, San José State University, San Jose, CA, United States

**Keywords:** creativity, personality, language use, science, art, assessment

## Abstract

The purpose of this study was twofold: first, to be among the first attempts to validate linguistic analysis as a method of creativity assessment and second, to differentiate between individuals in varying scientific and artistic creativity levels using personality language patterns. Creativity is most commonly assessed through methods such as questionnaires and specific tasks, the validity of which can be weakened by scorer or experimenter error, subjective and response biases, and self-knowledge constraints. Linguistic analysis may provide researchers with an automatic, objective method of assessing creativity, and free from human error and bias. The current study used 419 creativity text samples from a wide range of creative individuals mostly in science (and some in the arts and humanities) to investigate whether linguistic analysis can, in fact, distinguish between creativity levels and creativity domains using creativity dictionaries and personality dimension language patterns, from the linguistic inquiry and word count (LIWC) text analysis program. Creative individuals tended to use more words on the creativity keyword dictionaries as well as more introversion and openness to experience language pattern words than less creative individuals. Regarding creativity domains, eminent scientists used fewer introversion, and openness to experience language pattern words than eminent artists. Text analysis through LIWC was able to partially distinguish between the three creativity levels, in some cases, and the two creativity domains (science and art). These findings lend support to the use of linguistic analysis as a partially valid assessment of scientific and artistic creative achievement.

## The Language Of the Creative Person: Validating the Use of Linguistic Analysis to Assess Creativity

The track record of our species is filled with a myriad of creative accomplishments, some as grand as the great pyramids of Egypt and others as simple, yet significant, as the wheel. Both survival and mundane obstacles have been overcome with creative solutions. As Edward de Bono said, “there is no doubt that *creativity is the most important human resource of all*. Without creativity, there would be no progress and we would be forever repeating the same patterns” de Bono (1992, p. 169, emphasis added). Our survival and progress as a species thus far are partly due to our ability to be creative.

Understanding the importance of creativity and humanity’s dependence on it, researchers have been studying the creative person, process, and product. Traditionally, creativity is assessed through questionnaires and tasks, methods that require human raters; however, creativity has seldom been successfully assessed automatically through computerized programs. Linguistic analysis provides the opportunity to assess creativity both directly and through personality dimensions. If linguistic analysis proves to be a valid form of creativity assessment, linguistics and personality psychology will be able to make great strides in further creativity research. A major goal of the current study was to analyze the creative personality in science and art using linguistic analysis to determine if this approach provides a valid and relatively novel assessment of scientific and artistic creative achievement.

### Defining Creativity

Most of the contemporary definitions of creativity have the same criteria and are therefore, similar to one another (Newell et al., 1962; Stein, 1974). Runco and Jaeger (2012) explained that for something to be creative, two elements are required: originality, or what some people might refer to as novelty or uniqueness, and effectiveness, which in creativity may go by another name, such as “usefulness, fit, or appropriateness” (Runco and Jaeger, 2012, p. 92). Elaborating on the element of usefulness, Stein (in Taylor, 1964) clarified that something meets the criterion of being creative if, at some point in time, the product of the creative action or work results in something that is satisfying or useful to a group. However, Feist (2017) defines the second component of creativity not simply as usefulness, but rather meaningfulness: “to be classified as creative, thought or behavior must also have meaning to other people” (p. 186). This component of meaningfulness allows for a distinction between creativity and original nonsense (things that are simply novel but have no meaning).

#### Levels of Creativity

To examine whether linguistic analysis predicts variation in creative achievement it is important to understand that creativity is not a “yes-no” phenomenon, but rather exists on a continuum. To this point, creativity researchers have argued for at least four distinct levels of creativity, namely mini-c, little-c, Pro-C, and Big-C (cf. Csikszentmihalyi, 1998; Kaufman and Beghetto, 2009). The levels of creativity, from mini to Big, boil down to the range of their impact and influence over time and space. Big-C creative achievement, at its most extreme, consists of a contribution that lasts over centuries and across-nations if not continents (Simonton, 2017). That standard is extremely rare and would leave out nearly everyone if that were the threshold. Instead, a more reasonable criterion of Big-C creative achievement is work that currently has international impact and/or has started new fields or technologies. For Big-C to happen, the person, social system, and cultural system have to all agree the product is novel and meaningful. By this standard being elected a member of National Academy of Sciences or winning a Nobel prize would meet that criterion of Big-C. Pro-C, or professional-creativity, exists when one’s level of impact reaches primarily the regional or national level and/or is practiced by professionals who are not yet internationally eminent. But being professional by itself does not guarantee Pro-C creativity status (Kaufman and Beghetto, 2009). Little-c creativity describe creative works that have everyday, local/regional impact. If the social or cultural systems fail to appreciate the achievement, we have personal or little-c creativity. Lastly, mini-c creativity is highly personal and developmental (e.g., a high-school student taking an art class). For purposes of the current study, we argue that small-c creativity can be considered as a combination of the two lower levels of creativity: mini-c and little-c, the personal and everyday creativity of which everyone is capable.

#### Domains of Creativity

Within creativity and creative individuals, there are notable differences that have allowed for a division to be recognized within creativity creating different domains, or cultures, if you will. In his book, the *Two Cultures*, published in 1959, C. P. Snow was among the first to describe the conflict that exists in academia between the “two cultures” – the humanities and the sciences. Each culture holds specific views and impressions about the other: scientists believe that literary intellectuals have a complete lack of foresight and are unconcerned with their fellow humans while non-scientists believe that scientists are “shallowly optimistic” and are unaware about humans’ true condition. Snow (1959) maintained that while there does not appear to be a place where the cultures meet, “the clashing point of two subjects, two disciplines, two cultures – of two galaxies, so far as that goes – ought to produce creative chances” (p. 16). It is from these two cultures that great artists and scientists emerge and whose creativity and endeavors are “cornerstones of culture and provide mileposts of our cultural development and progress” (Feist, 2010, p. 113).

### Creativity and Personality

Over the last 30 years, many investigators have examined the personality qualities of creative people (Eysenck, 1995; King et al., 1996; Feist, 1998; Merten and Fischer, 1999; Wolfradt and Pretz, 2001; Dollinger et al., 2004; Silvia et al., 2008; Fürst et al., 2014; Isaksen et al., 2014; Karwowski and Lebuda, 2016; Kaufman et al., 2016; Lee and Min, 2016; Chiang et al., 2017; Puryear et al., 2017; Wang et al., 2017; Dumas et al., 2020). The strongest and most robust personality trait associated with creative achievement is Openness to Experience, which consists of a disposition to explore, to be curious, and to try a variety of experiences and sensations. Low Agreeableness/high hostility and impulsivity, also consistently covary with creative thought and behavior. That is, creative people tend to be socially aloof, challenge norms, be arrogant, and sometimes even hostile. In an early meta-analysis of creativity and personality in the scientific and artistic domains, Feist (1998) also reported that that Openness to Experience is the largest and strongest consistent predictor of creativity (cf. Karwowski and Lebuda, 2016). When looking at the relationship between extraversion and creativity, extraversion must be broken down into its two main components: sociability and dominance. Creative individuals are high in dominance and low in sociability. Feist (1998) also found that agreeableness and neuroticism have a negative relationship with creativity while also having the smallest effects. The relationship between conscientiousness and creativity is moderate; yet, the direction of the relationship is domain-dependent. In the artistic domain, conscientiousness is negatively related to creativity while in the scientific domain, it is positively related to creativity. Feist’s meta-analysis (1998) revealed that creative people tend to be more autonomous, introverted, open to new experiences, norm-doubting, self-confident, self-accepting, driven, ambitious, dominant, hostile, and impulsive compared to less creative people. Other non-Big Five models of personality have reported the personality traits associated with a high creativity index and high creative achievement are high exploratory excitability, low harm avoidance, high persistence, and high self-directedness and cooperativeness (Chávez-Eakle et al., 2006).

This review has only touched the surface, but it should be clear that there is a developed and rich empirical literature on creativity and personality. One topic, however, that has not been investigated is linguistic style and creativity – the main focus of the current study.

### Linguistic Analysis

Creative thought and behavior historically have been assessed either via divergent thinking tasks such as Torrance tests of creative thinking, the consensual assessment technique, or self-reports such as the creative achievement questionnaire (Amabile, 1996; Carson et al., 2005; Silvia et al., 2008). The language that people use in daily life also can be extremely revealing of one’s underlying psychology as there are connections between the style and content of an individual’s language and how one feels, thinks, and behaves (Boyd, 2017). Linguistic analysis, therefore, provides researchers the opportunity to explore psychological properties, such as creative thought, using a reliable method.

#### Language Use

The most widely used linguistic analysis program in the social sciences is linguistic inquiry and word count (LIWC) (Pennebaker et al., 2015a). LIWC is a computer-based text analysis program that provides users with frequencies (the percentages of total words in the text sample) in the output variables by analyzing the cognitive, emotional, and structural elements present in individual text samples by processing target words and matching them to internal dictionary words that tap into particular domains (Pennebaker et al., 2015b).

Inguistic inquiry and word count analyzes both content words, which communicate some kind of meaning, like who, what, where, or why (nouns, regular verbs, adjectives, and adverbs), and function or style words (pronouns, prepositions, auxiliary verbs, and conjugations, etc.) that are used to link meaningful words together, which are generated from a deep level of the mind and are often automatic and used unconsciously, consequently revealing an individual’s psychological state (Tausczik and Pennebaker, 2010; Boyd, 2017). The advantage of LIWC’s word-counting approach for exploring the psychological processes found in individuals’ language is that the reliability of LIWC’s results is never undermined by experimenter error or subjective bias (Ireland and Mehl, 2014).

Inguistic inquiry and word count, however has seldom been used to assess an individual’s level of creativity or their creative ability (Kelley et al., 2019; it has primarily been used with creativity when participants are asked to provide a creative writing sample, which is then used to assess other constructs, such as work-life narrative, emotional coping, and motivation (Djikic et al., 2006; Lengelle et al., 2013; Kelley and Ireland, 2017). The current study will examine whether or not linguistic style and content can differentiate creative from less creative people.

#### Language Use and Personality

It can be problematic to rely on self-report questionnaires as the “gold standard” scores for personality research because of potential response biases and self-knowledge constraints (Paulhus and Vazire, 2007). Linguistic analysis has become a technique for personality researchers to assess personality in a less biased and more reliable way (Yarkoni, 2010; Ireland and Mehl, 2014; Obschonka et al., 2017; Kern et al., 2019). A more “psychologically telling” and psychometrically parsimonious method of determining individual differences is language styles or *how* an individual says things, rather than differences in language content or *what* an individual says (Yarkoni, 2010; Ireland and Mehl, 2014).

Researchers have reported consistent relationships between linguistic style and the Big Five elements of personality (Pennebaker and King, 1999; Mairesse et al., 2007; Yarkoni, 2010; Iacobelli et al., 2011; Ireland and Mehl, 2014) (see Table 1). Researchers have found that introverts use more articles, exclusive words, negations, and tentative words – categories that result in a more concrete and descriptive language style that is careful, precise, and focused, compared to extraverts who have a more abstract and interpretive language style (Pennebaker and King, 1999; Nowson, 2006; Oberlander and Gill, 2006; Beukeboom et al., 2012). Individuals high in Openness to Experience, compared to those low in Openness, tend to express positive feelings and use articles, longer words, insight words, and inclusive words (Pennebaker and King, 1999; Nowson, 2006). Openness is strongly related to greater use of perceptual processes, which include words related to seeing and hearing (Hirsh and Peterson, 2009).

**TABLE 1:** Personality dimension language use patterns.

Personality dimension	LIWC categories	Examples
Introversion	Articles	a, an, and the
	Negations	no, never, and not
	Negative emotions	hate, worthless, and enemy
	Causation	because, effect, and hence
	Discrepancy	should, would, and could
	Tentative	maybe, perhaps, and guess
	Differentiation	but, except, and without
	Body	ache, heart, and cough
	Achievement	try, goal, and win
	Fillers	blah, you know, and I mean
Openness to experience	Articles	a, an, and the
	Past tense	walked, were, and had
	Prepositions	with and above
	Positive emotions	happy, pretty, and good
	Social processes	talk, us, and friend
	Tentative	maybe, perhaps, and guess
	Conjunction	with, and, and include
	Seeing	view, saw, and look
	Sexuality	horny, love, and incest
	Leisure	house, TV, and music
	Religion	altar, church, and mosque
	Death	bury, coffin, and kill
	Swear words	*****

It is important to note that as of the 2015 version of LIWC, the Exclusive and Inclusive word categories have been changed to the Differentiation and Conjunction categories, respectively, due to “weak” and “terrible” psychometrics (Pennebaker et al., 2015a).

#### Language Use and Creativity

There has not been much research examining language use and creativity, specifically the language used in describing creative work and the language used by highly creative individuals. Four exceptions to this trend are research by Pennebaker and Stone (2003); Borowiecki (2017), Kelley and Ireland (2017), and Kelley et al. (2019). Pennebaker and Stone (2003) used LIWC to explore the relationship between aging and language use for over 3,000 research subjects from 45 different studies as well as the collected works of 10 eminent poets, novelists, and playwrights from the last 500 years. They found that as individuals age, they use fewer self-reference, past-tense, and negative affect words and more future-tense and positive affect words, all while exhibiting a pattern of increasing cognitive complexity. Borowiecki (2017) explored the relationship between negative emotions and creativity using LIWC to analyze 1,400 letters written by three eminent composers: Wolfgang Amadeus Mozart, Ludwig van Beethoven, and Franz Liszt. He explored the association between negative emotions and outstanding creative achievements and found that creativity is causally attributed to negative states, particularly sadness. Kelley and Ireland (2017) used LIWC to explore nearly 1,500 artists’ potential motivations for writing from the artists’ writings on art practice, artwork, art movement, artists, curators, patrons, and critics. They found that artists use words higher in cognitive complexity and meaning-making while having a high drive for achievement and low social affiliation and connectivity (Kelley and Ireland, 2017). Finally, Kelley et al. (2019) also used LIWC to explore whether or not Intellect can predict high achievement of visual artists using over 2,000 writing samples of visual artists and scientists. There were no meaningful differences across the linguistic categories associated with Intellect between eminent artists and scientists; therefore, Intellect is equally associated with eminent creative achievements in the arts and the sciences.

Inguistic inquiry and word count dictionaries can be used to identify creativity language use patterns. Toward this end, a Creativity and Innovation Dictionary for LIWC was created by Neufeld and Gaucher in 2017 (see Table 2). The Creativity and Innovation LIWC Dictionary was created through multiple rounds of synonym collection for the words “creativity” and “innovation” from dictionaries and thesauri. Each word was assessed to determine whether it was a conceptual match to the original words and whether it had any other non-creativity or non-innovation synonyms. The words that were a conceptual match and did not have any undesirable synonyms were included in the creativity and innovation dictionary resulting in the final dictionary consisting of 86 words (Neufeld and Gaucher, 2017).

**TABLE 2:** Creativity and innovation LIWC dictionary (Neufeld and Gaucher, 2017).

Actualiz*	Expand*	Innovate*	Radical
Adapt*	Device*	Inspire*	Resourceful*
Advanc*	Devis*	Introduce*	Revolution*
Artistic	Differ*	Invent*	Set up
Avant-garde	Discover*	Lead*	Shift*
Best-in-class	Experiment*	Leading-edge	Solv*
Brainstorm*	Forge	Metamorphosis	Spawn*
Build*	Form*	Modern*	State-of-the-art
Change*	Found*	Modif*	Surpris*
Clever*	Fresh*	New*	Trailblaz*
Conceiv*	Future	Novel*	Transform*
Contemporary	Generat*	Odd*	Uncommon
Craz*	Ground-breaking	Offbeat	Unfamiliar*
Create*	Grow*	Open-mind*	Unique*
Cutting-edge	Hatch*	Opportunity*	Unprecedent*
Depart*	Imagin*	Origin*	Unusual*
Design*	Improv*	Peculiar	Unveil*
Develop*	Individual*	Pioneer*	Upheav*
Enhanc*	Industry-leading	Problem-solv*	Vicissitude*
Enterprising	Ingen*	Produc*	Vision*
Efficien*	Initiat*	Prolific	Wild

*Word stem.

Jordanous (2012) created a list of the “Top 100 creativity corpus keywords,” which is a list of keywords for creativity (see Table 3). Although the list is not an explicit creativity dictionary, like Neufeld and Gaucher’s (2017), the list Jordanous created is valuable for evaluating creative practices and exploring the nature of creativity. Jordanous (2012) explored the relationship between creativity words used by academic scholars who study the creative person and process and general academic words used in written English (found in the Academic Word List and the University Word List) and was left with a list of 694 words (389 nouns, 205 adjectives, 72 verbs, and 28 adverbs). The top 100 words in the list are valuable for linguistically assessing creativity as they are the “keywords that highlight key components of creativity” (Jordanous, 2010, p. 279).

**TABLE 3:** Top 100 creativity corpus keywords (Jordanous, 2012).

Creative	Artistic	Unconscious
Creativity	Evolutionary	Probability
Cognition	Correlated	Self
Domain	Ability	Knowledge
Innovation	Programs	Variables
Openness	Intelligence	Primitive
Because	Cannot	Novelty
Divergent	Facilitate	Subjects
Process	Toward	Retention
Motivation	Correlation	Dimensions
Domains	Basis	Hypotheses
Found	Computational	Innovative
Abilities	Extrinsic	Ideas
Thinking	Selective	Related
Scores	Cognition	Dimension
Solving	Hypothesis	Validation
Individuals	Interactions	Attributes
Personality	Criterion	Research
Scales	Validity	IQ
Processes	According	Artifacts
Empirical	Measures	Combinations
Ratings	Tests	Predictions
Correlations	Verbal	Heuristic
Originality	Investigations	Factors
Traits	Heuristics	These
Associative	Fluency	Psychology
Influences	Rated	Barren
Primary	Psychologists	Positively
Conceptual	Complexity	Investigators
Instance	Discoveries	Perceptual
Developmental	Semantic	Example
Individual	Discovery	Elements
Problem	Schema	
Intrinsic	Rat	

## Current Study

The purpose of the current study was to be among the first to examine the idea that linguistic analysis can provide validation for distinguishing individuals high in scientific and artistic creativity from those lower in it, as well as for understanding the personality-related language use patterns of Big-C, Pro-C, and Small-c individuals. Because there is very little research examining the direct relationship between creativity and language use patterns, this study used personality-related language use patterns to examine the relationship between creativity and linguistic style.

Linguistic analyses were conducted using the LIWC program and statistical analyses were conducted using SPSS-26. Interviews from Gregory Feist’s dissertation Feist’s (1991) and Lisl Marburg-Goodman’s book *Death and the Creative Life* (1981) as well as lectures of selected Nobel Laureates and selected blogs were analyzed using LIWC. The hypotheses of the current study were:

(1). Individuals who have achieved Big-C creativity will use more words from the Creativity and Innovation LIWC Dictionary (Neufeld and Gaucher, 2017) than those classified as Pro-C, who in turn will use more creativity and innovation words than people classified as Small-c creativity after controlling for mode of language.
(2). Individuals who have achieved in the Big-C creativity will use more words from the creativity corpus keywords (Jordanous, 2012) than those classified as Pro-C, who in turn will use more creativity corpus keywords than and people classified as Small-c creativity after controlling for mode of language.
(3). Individuals who have achieved Big-C creativity will use more Introversion Language Pattern words than those classified as Pro-C, who in turn will use more introversion words than people classified as Small-c creativity after controlling for mode of language.
(4). Individuals who have achieved Big-C creativity will use more Openness to Experience Language Pattern words than those in the Pro-C, who in turn will use more creativity corpus keywords than and people classified as small-c creativity after controlling for mode of language.
(5). Big-C scientists will use less Introversion Language Pattern words than Big-C artists after controlling mode of language.
(6). Big-C scientists will use less Openness to Experience Language Pattern words than Big-C artists after controlling for mode of language.

In sum, this study examined whether linguistic analysis is a valid or invalid form of assessing creativity levels and domains. By using interviews and public lectures, we attempt to validate linguistic analysis as a method of creativity assessment. Blog entries and interviews of less-creative individuals served as the comparison to more-creative individuals and to further validate the linguistic analysis. If the results suggest that linguistic analysis is a valid form of assessment, then it will be a relatively novel and efficient method of assessing creativity as it will eliminate the need for human involvement in the scoring process.

## Materials and Methods

The current study was archival and involved analyzing texts written and spoken by a range of creative levels and domains. The texts analyzed in this study came from four different sources: *Death and the Creative Life* (Marburg-Goodman, 1981), Gregory Feist’s dissertation interviews Feist’s (1991), Nobel Laureate Lectures, and blogs from the internet. A total of 419 text samples across all sources were used in this study (see Table 4). Demographics from individuals whose language samples were used were collected and compiled. The demographics collected were gender and nationality. However, demographics were not available for all subjects. Gender was coded as either male or female, nationality was coded as either single, dual, or multiple nationality, and mode of language was coded as either written or spoken.

**TABLE 4:** Subjects.

Group	*n*	Gender		Creativity Level			Nationality		
				
		Male	Female	Big-C	Pro-C	Small-c	Single	Dual	Multi
Nobel Laureate	249	239	10	249	0	0	187	57	5
Physics	55	55	0	55	0	0	42	10	3
Chemistry	60	59	1	60	0	0	45	14	1
Medicine	58	57	1	58	0	0	43	14	1
Literature	42	35	7	42	0	0	30	12	0
Economic Sciences	34	33	1	34	0	0	27	7	0
Marburg-Goodman	28	27	1	22	2	4	11	9	1
Scientists	11	11	0	11	0	0	6	2	1
Artists	11	10	1	11	0	0	4	7	0
Unfulfilled	6	6	0	0	2	4	1	0	0
Feist	99	99	0	31	68	0			
Physics	29	29	0	9	20	0			
Biology	28	28	0	10	18	0			
Chemistry	42	42	0	12	30	0			
Blog	43	21	21	0	32	11			

*N = 419.*

The Small-c creativity level consisted of individuals in the mini-c or little-c creativity level. This included career fields that did not require creativity. The Pro-C creativity level consisted of individuals whose careers required creativity. The Big-C creativity level consisted of individuals who have reached eminent creative status, whether by accomplishment or recognition.

### Sources of Texts

#### Big-C Sample

Twenty-two interviews from Marburg-Goodman’s book *Death and the Creative Life* (1981) were taken to be a part of the Big-C sample for the study. The Big-C sample from this source consisted of eminent creatives from two domains, art (*n* = 11) and science (*n* = 11). Thirty-one interviews of scientists from Feist’s dissertation Feist’s (1991) were taken to be a part of the Big-C sample for the study. To qualify as part of the Big-C sample, the criteria of eminence for the scientists in Feist’s sample was that they must be members of the National Academy of Sciences. The Big-C sample from this source consisted of scientists from the three major scientific disciplines: biology (*n* = 10), physics (*n* = 9), and chemistry (*n* = 12).

The third source of the Big-C sample came from Nobel Laureates. Nobel Lectures were taken from each of the five categories of Nobel Prizes: physics, chemistry, medicine, literature, and economic sciences. The lectures were taken from the Nobel Prize website^1^ and were chosen based on their content and whether or not they were told in a story-like fashion and from a first-person perspective. The Nobel Prize and the Prize in Economic Sciences have been awarded 597 times. This was the initial subject pool. However, because there were laureates who had not given a lecture or had not presented it from a first-person perspective in a story-like manner, the number of Nobel Lectures used in this study was 248. Fifty-five Nobel Laureates’ lectures were chosen from Physics Prize winners, 60 Nobel Laureates’ lectures were chosen from the Chemistry Prize winners, 58 Nobel Laureates’ lectures were chosen from the Medicine Prize winners, 42 Nobel Laureates’ lectures were chosen from the Literature Prize winners, and 34 Nobel Laureates’ lectures were chosen from the winners of the Prize in Economic Sciences.

#### Pro-C Sample

The Pro-C sample consisted of individuals whose profession required creativity, but who had not yet reached internationally eminent status through their work. One interview of an “Unfulfilled” individual from Marburg-Goodman’s book was taken to be a part of the study in the Pro-C sample. This particular interviewee had a career that fell under engineering.

Sixty-eight scientists’ interviews from Feist’s dissertation Feist’s (1991) were taken to be a part of the Pro-C sample of the study. The scientists in the Pro-C sample are creative and relatively eminent (full professors at major research universities) but not eminent as defined by being members of the National Academy of Sciences. The Pro-C sample from this source consisted of scientists from the three major scientific disciplines: biology (*n* = 18), physics (*n* = 20), and chemistry (*n* = 30).

The third source of the Pro-C sample came from bloggers. A list of professions was created after searching for different types of professions on Google.com. With a compiled list of professions, blogs were then found by searching “diary of a [profession]” and “[profession] blogs” on Google.com for each profession from the list. The selection criteria for the blogs were that they must be told from a first-person point of view rather than a third-person point of view and be about the blogger’s profession. The blogger’s follower-base size was not considered or used in the selection process because the blog’s impact or influence on others was not a criterion as the blogs were meant to be the less-creative Small-c sample. Using this selection criteria, 43 blogs, and subsequently 43 blog posts, were selected to serve as text samples for this study. Thirty-two bloggers fell under the criteria of being in the Pro-C creativity level in that they were earning money from their profession. The career fields represented in the Pro-C blog samples were biological sciences (*n* = 16), psychology (*n* = 1), engineering (*n* = 3), art (*n* = 3), literature (*n* = 4), architecture (*n* = 2), and culinary (*n* = 3).

#### Small-C Sample

As a comparison group for the more creative samples, Marburg-Goodman’s (1981) interviews of the “Unfulfilled” and blogs from everyday professions were used for the Small-c sample. Five interviewees from the “Unfulfilled” group from Marburg-Goodman’s book were taken to be a part of the study in the Small-c sample. The career fields represented by the five interviewees were banking (*n* = 1), stocks (*n* = 1), teaching (*n* = 1), and unemployed or unknown (*n* = 2).

The search for blogs of Small-c individuals followed the same method as the Pro-C blogs. From the list of 43 blogs, 11 belonged to Small-c individuals. The career fields represented in the Small-c blog samples were public service (*n* = 6), trade (*n* = 1), agriculture (*n* = 2), and beauty (*n* = 2). The interviews of Marburg-Goodman’s “Unfulfilled” (1981), along with the blog posts and LIWC norms from the literature, served as comparison groups against the Big-C and Pro-C creativity samples.

### Text Cleaning

All texts were cleaned so that only what the interviewees, Nobel Laureates, and bloggers said or wrote were left in the text files. Texts from the interviewers as well as quotes, poems, charts, graphs, images, and equations, were scratched from each text sample file. A folder containing all 419 text samples was uploaded into LIWC and run through each category of the 2015 LIWC dictionary, excluding the punctuation and net speak categories.

### Creativity and Personality Dictionaries

The text files were run through the Creativity and Innovation Dictionary (Neufeld and Gaucher, 2017), and the LIWC dictionary that was made from the top 100 creativity key words compiled by Jordanous (2010) in her creativity corpus (also called the creativity corpus keywords dictionary in this study), and the personality language use dictionaries. For example, word stems such as “artistic,” “clever,” “ingen,” and “innovate” make up the creativity/innovation dictionary (see Table 2).

The second creativity dictionary was based on the research of Jordanous (2012), who wanted to obtain unique creativity words that scholars of creativity and the creative process use when discussing creativity. She did this by obtaining words from the text of 30 classic scientific papers on creativity, the creative process, and the creative person published between 1950 and 2009. Then she selected words using a *G*^2^ statistic that is analogous to χ^2^, in that it is an index of expected frequency of word use in the English language compared to its observed frequency of word use in the creativity literature. The higher the *G*^2^ score the more unique it is to the creativity literature compared to written language in general. Her initial search obtained 694 words more frequently used in creative academic papers than in written English texts in general. She then took the top 100 of these 694 words and that became the list of creativity keywords, the top 10 of which were: *creative, creativity, cognitive, domain, innovation, openness, because, divergent, process, and motivation* (see Table 3). Finally, the lead author (STA) used these 100 keywords to create a new LIWC dictionary for the current study.

The personality language use dictionaries for Introversion and Openness to Experience were made from words that represented language in a personality space (Schwartz et al., 2013), and the words that fell under the LIWC categories correlated with Introversion and Openness to Experience (see Table 1; Mairesse et al., 2007; Yarkoni, 2010; Iacobelli et al., 2011; Ireland and Mehl, 2014). These dictionaries have validated the LIWC dimensions with the Big Five dimensions of personality.

## Results

The purpose of the first two analyses was to validate the Creativity and Innovation LIWC Dictionary (Neufeld and Gaucher, 2017) and the creativity corpus keywords dictionary (the top 100 creativity keywords from the creativity corpus) (Jordanous, 2012) and examine whether there were creativity word differences between the three levels of creativity. The purpose of the third analysis was to explore personality language patterns (introversion and openness) and creativity levels, whereas the fourth analysis was to explore personality language pattern differences between Big-C scientists and artists.

**FIGURE 1:**
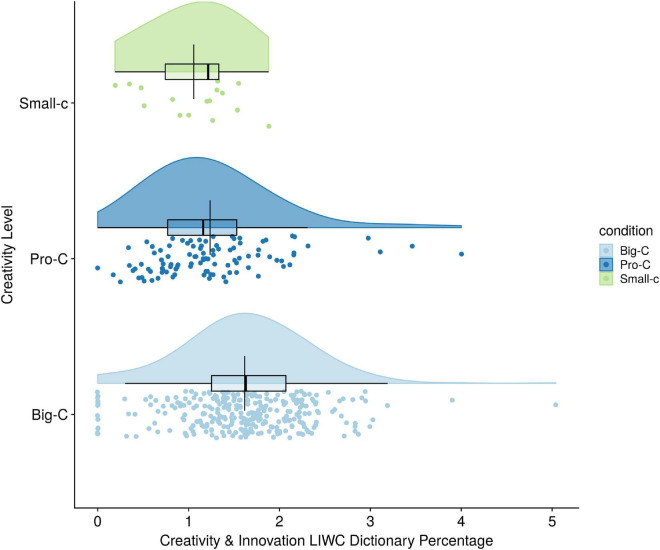
Creativity level differences on creativity and innovation LIWC dictionary frequency percentages.

The first two hypotheses were validity checks of the creativity word dictionaries to see whether the most creative people used more creativity words than the less creative people. Hypothesis 1 was that individuals in the Big-C creativity level would use more words from the creativity and innovation LIWC dictionary than subjects in the Pro-C, who in turn would use more such words than Small-C creativity participants. Because type of language mode [spoken (0) vs. written (1); *r* = 0.30] covaried with Creativity/Innovation words, it was added as a covariate in the one-way ANCOVA. Results of the evaluation for normality and homogeneity of variance assumptions were satisfactory. Results of the ANCOVA showed no overall differences in creativity/innovation words percentage between the 3 creativity levels (see Figure 1). As seen in Table 5, there were no mean differences between the three levels of creativity on creativity and innovation word use percentage [*F*(2,414) = 1.08, ns, partial *i*^2^ = 0.005]. None of the three simple comparisons between each group was significant.

**TABLE 5:** Means, standard deviations for Big-C, Pro-C, and Small-c samples on creativity and personality words percentage (Hypotheses 1–4).

	Big-C		Pro-C		Small-c	
			
	(*n* = 302)		(*n* = 101)		(*n* = 16)	
			
Variable	*M*	*SD*	*M*	*SD*	*M*	*SD*
Creativity and innovation LIWC	1.61	0.69	1.24	0.67	1.06	0.48
Creativity corpus keywords	1.21	0.56	1.35	0.73	0.65	0.38
Introversion language patterns	22.13	3.26	23.71	3.47	23.67	4.06
Openness language patterns	44.25	5.36	49.58	3.15	46.4	5.57

Hypothesis 2 was that individuals in the Big-C creativity level would use more words from the creativity corpus keywords than subjects in the Pro-C, who in turn would use more creativity words than Small-c creativity participants. Because type language mode [spoken (0) vs. written (1); *r* = 0.22] covaried with creativity corpus keywords, it was added as a covariate in the one-way ANCOVA. Tests of assumptions (normality and homogeneity of variance) showed no violations. There were only three univariate outliers from the Nobel Laureate and Marburg-Goodman groups.

**FIGURE 2:**
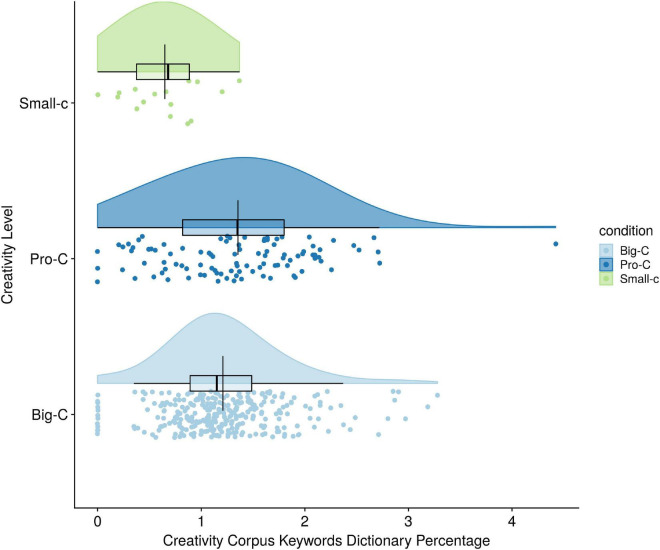
Creativity level differences on creativity corpus keywords frequency percentages.

After holding mode of language constant, there was an overall difference between the three creativity levels on creativity corpus keywords percentages [*F*(2,414) = 10.63, *p* < 0.001, partial *i*^2^ = 0.05; see Table 5 and Figure 2]. Creativity level explained 5% of the variance in creativity corpus keywords dictionary percentage. This result suggests creativity corpus keywords are valid assessments of creativity level. Bonferroni comparisons (with alpha levels of.017) revealed that the Pro-C and Big-C creativity groups had statistically higher percentages of creativity corpus keywords dictionary usage than the Small-c creativity group (*p*-values of.001 and.000 respectively). Turning to personality language use and creativity, Hypotheses 3 and 4 were that individuals in the Big-C creativity groups would use more Introversion Language Pattern and Openness to Experience Language Pattern words, respectively, than those in the Pro-C and Small-c creativity groups, after controlling for mode of language. Hypotheses 3 and 4 were tested with a one-way analysis of covariance (ANCOVA) (see Table 5). The independent variable was creativity group, defined categorically as Small-c, Pro-C, and Big-C, and the two dependent variables were Introversion Language Patterns (Hypothesis 3) and Openness to Experience Language Patterns (Hypothesis 4). Tests of homogeneity of variance and equality of covariance matrices were met. In addition, 14 multivariate outliers from the Nobel Laureate, Marburg-Goodman, and blog groups were found using the Mahalanobis distance test (cases having a critical value over 13.82 were considered multivariate outliers). After the removal of the 14 outlier cases, neither output variable, introversion language patterns or openness to experience language patterns, was skewed.

**FIGURE 3:**
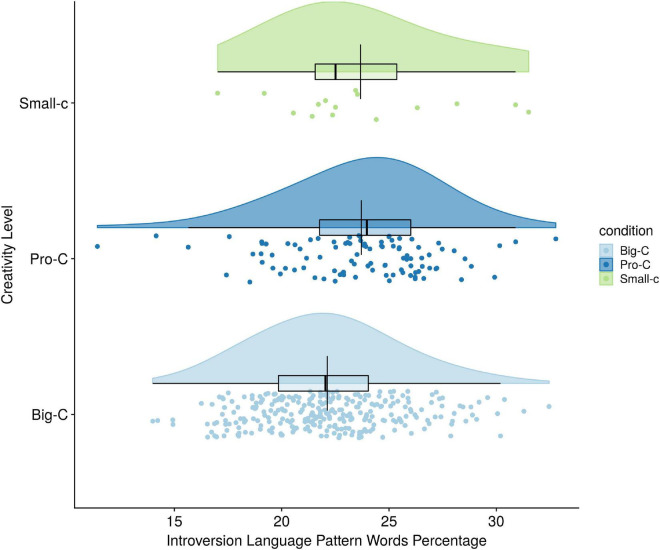
Creativity level differences on introversion language pattern use.

The ANCOVA test of Hypothesis 3 revealed that there was no overall difference between the three levels of creativity on their percentage of Introversion words usage after language mode was held constant [*F*(2,401) = 1.98, *p* = *0.14*; partial η*^2^* = 0.01]. In other words, only 1% of introversion language pattern usage was attributable to creativity level. Because the omnibus ANCOVA was not significant, no further analyses were conducted (see Figure 3).

**FIGURE 4:**
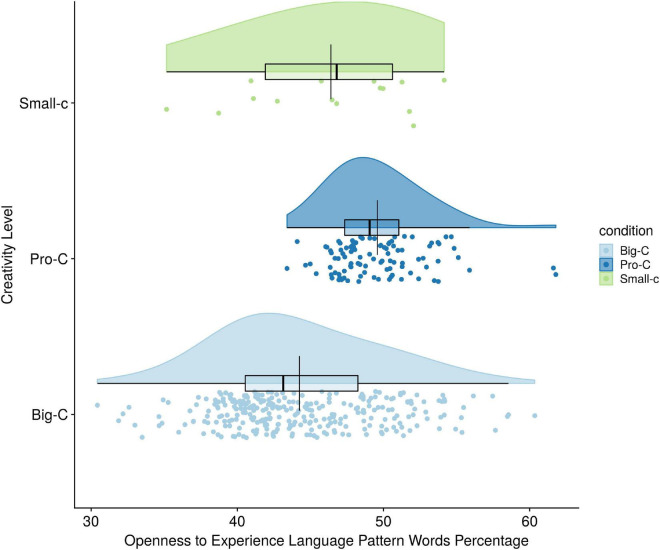
Creativity level differences on openness to experience language pattern use.

The one-way ANCOVA for Hypothesis 4, however, revealed a significant overall difference in percentage of openness language use between the three creativity levels [*F*(2,401) = 33.09, *p* < 0.001, η*^2^* = 0.142]. Holding language mode constant, creativity level explained 14% of the variance in openness language use. Bonferonni comparisons showed the only specific comparison that was significant was the Big-C/Pro-C comparison, with Pro-C using significantly more openness words than the Big-C group (*p* < 0.001). The Small-c/Pro-C and Small-c/Big-C comparisons showed no group differences in use of Openness words (see Figure 4).

Hypotheses 5 and 6 predicted that Big-C scientists would use fewer introversion language pattern and fewer openness to experience language pattern words, respectively, than Big-C artists after controlling for mode of language. These hypotheses were tested with a one-way ANCOVA (see Table 6). Tests of the equality of covariance matrices and homogeneity of variances assumptions were satisfactory.

**TABLE 6:** Descriptive statistics for language pattern words percentage of Big-C scientists and artists (Hypotheses 5 and 6).

Variable	Big-C group	*n*	Mean	*SD*
Introversion language patterns	Scientists	180	19.76	4.68
	Artists	53	23.85	2.68
Openness language patterns	Scientists	180	39.79	8.96
	Artists	53	50.00	5.97

**FIGURE 5:**
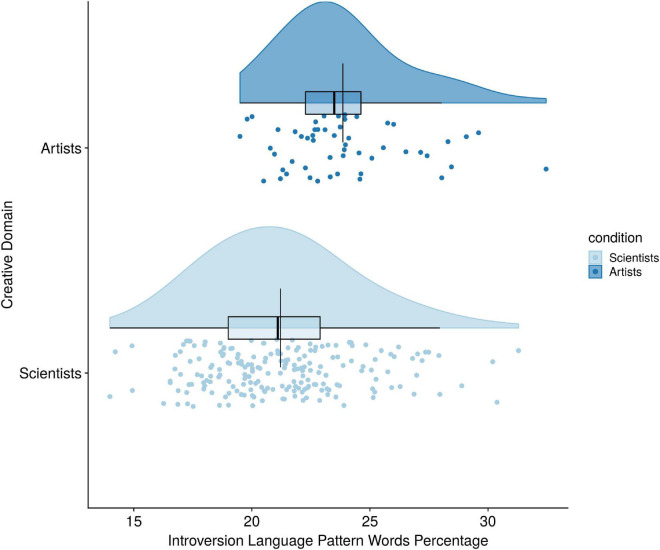
Creative domain differences on introversion language pattern use.

**FIGURE 6:**
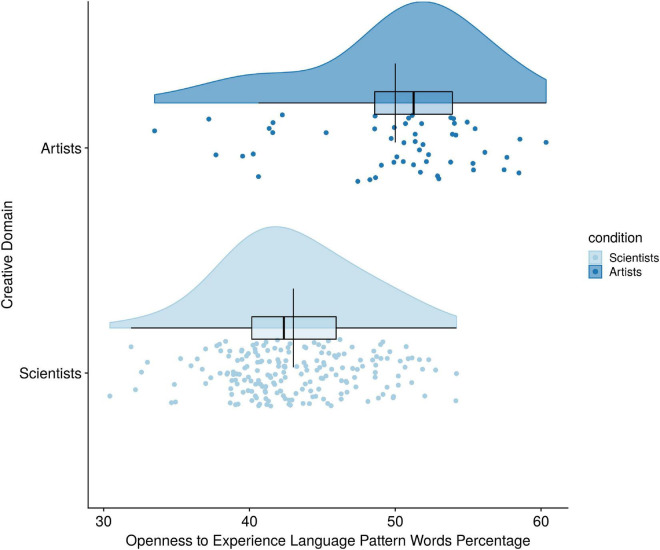
Creative domain differences on openness to experience language pattern use.

As predicted by Hypothesis 5, there was a statistically significant difference between Big-C Art and science domains on introversion language pattern percentages after mode of language was held constant [*F*(1,347) = 25.65, *p* < 0.001, partial η*^2^* = 0.069] (see Figure 5). Because there were only two groups, we can conclude the Big-C scientists used significantly fewer Introversion words than Big-C artists. Similarly, the test of Hypothesis 6 revealed that Big-C scientists also used significantly fewer openness language pattern words than Big-C artists after language mode was held constant [*F*(1,347) = 73.20, *p* < 0.001, partial η*^2^* = 0.174] (see Figure 6).

## Discussion

The purpose of this study was twofold: first, to be among the first attempts to validate linguistic analysis as a method of creativity assessment and second, to differentiate between individuals in varying scientific and artistic creativity levels using personality language patterns. Linguistic differences between creativity levels were explored using the Creativity and Innovation LIWC dictionary, creativity corpus keywords dictionary, and introversion and openness to experience language patterns. To explore the differences between eminent scientists and artists, linguistic analysis was conducted using introversion and openness to experience language patterns of LIWC.

The creativity word dictionaries were partially validated by the current results. Creative individuals used more creative language dictionary keywords than less creative people. Because the findings were not significant, Hypothesis 1 was not supported. The results of the analysis suggest that this particular dictionary does not discriminate between different levels of creative achievement. This may be because the creativity and innovation LIWC dictionary was created from synonyms of “creativity” and “innovation,” which may not be the language style of creative individuals. This dictionary also primarily consisted of content words rather than style words, which are more psychologically telling and more associated with personality.

When creativity levels were compared on creativity corpus keywords dictionary usage, however, Pro-C individuals and Big-C individuals used more creativity keywords than the Small-C sample. What is most interesting about this finding is that it suggests that highly creative scientists and artists speak about their work using language that is similar to the language used by creativity scholars to describe the creative person and process. Pro-C and Big-C samples could not be differentiated on the creativity corpus keywords dictionary. In sum, the results of the analysis demonstrate that the creativity corpus keywords dictionary is at least a partially valid assessment of creativity because of its ability to different real-world groups of varying degrees of creative achievement.

Additionally, openness to experience, but not introversion, language pattern words differentiated the groups but not always in the predicted directions. When creativity levels were compared on personality language pattern differences, the Big-C sample used significantly fewer introversion language pattern words in their text samples compared to Pro-c samples. These findings demonstrate that openness to experience language patterns are consistent with the findings from self-report, tasks, and consensual assessment techniques and that openness to experience (language) is a robust predictor of creative achievement. The surprise here is that professionally creative sample used more openness words than the Big-C sample. This finding requires replication and further study to explain why that might be.

Finally, in the last set of analyses we compared only Big-C scientists to Big-C artists and found differences in personality language. In particular, Introversion language patterns, Big-C scientists used significantly fewer introversion language pattern words in their text samples compared to Big-C artists. These findings demonstrate that introversion language patterns are a valid method of distinguishing between the creative domains of art and science. Likewise, Big-C scientists used significantly fewer openness to experience language pattern words in their text samples compared to Big-C artists. This finding suggests that openness to experience language patterns is a valid method of distinguishing between the creative domains of art and science.

### Implications

In sum, the two primary goals of the current study confirm that the creativity corpus keyword dictionary can at least at times differentiate creative from less creative people. Highly creative people tend to write and speak in ways similar to those who study creative people. The creativity and innovation dictionary, however, was not validated as a method of assessing creativity. Second, personality language use differentiates creative from less creative people as well as creative scientists from creative artists. It is important to remember that these findings stand after holding mode of language (spoken vs. written) and how multinational people were constant. Therefore, these findings remain regardless of whether the language was spoken or written or whether the speakers/writers were uni-, bi-, or multi-national. These findings suggest that linguistic analysis may be added to the arsenal of ways to assess creative people. In other words, researchers will no longer need to rely solely on previous measures of creativity, such as self-report questionnaires and tasks that are subject to scorer error, biases, and self-knowledge constraints.

### Limitations

As is true for all studies, this study has limitations. Perhaps the most obvious limitation is the uneven sample sizes for the creativity groups. The Small-c group in particular was unusually small and needs to be enlarged before the current findings can be confirmed.

Furthermore, overall there were fewer female subjects (*n* = 32) compared to male subjects (*n* = 385); a ratio of nearly 12 to 1. With more male subjects in every category (creativity level and domain), the gender differences in the population of both Big-C and Pro-C are great and highly skewed. These ratios are relatively representative of population differences. The question is, then, why are the population differences between genders so skewed? Perhaps these differences are due to the historical lack of female representation in highly creative fields, specifically in the sciences. This heavily male-dominated sample contributes to the lack of generalizability of these results since the results can only be generalized to creative male individuals and not the entire population of creative individuals.

Another limitation is that linguistic analysis was conducted using English language dictionaries, either from or uploaded to LIWC, on text samples taken from some subjects whose primary language was not English. Also, some of the Nobel Laureate lectures were written in different languages and then translated into English for accessibility. Having subjects whose primary language was not English and whose original words have been translated from another language can lead to a loss in meaning and words, weakening the validity of the linguistic analysis.

The two creativity language dictionaries used, the Creativity and Innovation LIWC Dictionary and the creativity corpus keywords dictionary, were mostly “creativity” and “innovation” synonyms as well as words related to research. Creative individuals do not speak saying “creative” or “innovative.” Rather they use words that demonstrate greater conceptual distances, reflecting their cognitive flexibility and divergent thinking. The words in these two dictionaries may not fully capture how creative individuals talk compared to less creative individuals, decreasing the internal validity of these dictionaries as methods to assess creativity linguistically. Perhaps a more valid and reliable dictionary would be one that is created not by finding synonyms of “creativity” and “innovation” or the most used words in the literature on creativity, but rather by gathering words used by highly creative individuals to describe their work. The dictionary approach is limited in its ability to assess creativity in text form as it is unable to capture the hallmarks of creativity: cognitive flexibility and divergent thinking as these are not represented in individual words stripped of their context.

In addition, LIWC, the linguistic analysis program used, is rigid in that it strictly understands only words and not context. This can lead to phrases being interpreted differently by the program from how the subject had intended his or her words to be interpreted. LIWC uses a closed approach using closed-vocabulary and word counting to analyze language. Perhaps a better method to analyze language is an open approach, which extracts comprehensive language features from text rather than relying on a priority word or category judgments (Park et al., 2015). Open approaches to language analysis have an advantage over closed approaches in that open approaches are able to accommodate neologisms and unconventional language use as well as extract many more and richer features from language samples (Park et al., 2015). A related limitation is that the only measure of personality used to distinguish between creativity levels and domains was introversion and openness to experience language patterns from the literature; no other measure of personality was used.

Method differences regarding the original setting and context of the text samples cannot be ruled out as a potential confound. Nobel Lectures are meant to be extremely formal, interviews are slightly less formal, and blogs are a very casual medium. Formality differences in the method–but not mode of transmission–pose as a possible confound because these differences in formality, rather than creativity level, may have resulted in differences in word usage and linguistic styles.

Despite the potential limitations noted, this study succeeded in its aim to investigate whether or not linguistic style can differentiate creative from less creative people and provide validation for distinguishing between creativity levels as well as creativity domains.

### Future Research

Future research could explore the use of introversion and openness to experience language patterns by creative individuals to better understand personality-specific linguistic styles. Similarly, affect, drives, and motivations should also be linguistically explored to gain more insight into the creative process. Future research can also explore linguistic differences between different fields within the creativity domains of art and science. Linguistic analyses should also be conducted in other languages, specifically the original language of texts, so that findings will have greater validity. For example, another method of automated text analysis, similar to LIWC, is computer-based analyses using Martindale’s innovative Martindale (1975, 1990) regressive imagery dictionary (RID). To assess regressive thought, the RID is composed of approximately 3,000 English words and stems in 29 categories. The RID was designed to distinguish between and measure primordial (associative, concrete, irrational, and dream-like) and conceptual (abstract, logical, and reality-orientated) thinking. In addition to using LIWC, future studies could utilize the RID when performing text analyses to gain further insight into creativity and primordial thinking (Kozbelt et al., 2014).

Alternatively, a better non-dictionary method of linguistic analysis for assessing creativity might be semantic distance. Semantic distance is a concept from psycholinguistic research and is essentially the number of steps that are between two concepts or words in semantic memory (Kenett, 2018). The associative theory of creativity is the main theory that connects semantic distance to creative thinking. In this theory, creativity is characterized by the association of weakly related and remote concepts into original and appropriate concepts (Kenett and Faust, 2019). The more creative a new combination of concepts is, the farther apart they are. Future studies should assess creativity using semantic distance to explore whether or not more creative individuals have greater semantic distance because their thoughts are more complex and more semantically distanced than those of less creative individuals.

Linguistic analysis is a newer, more efficient method of assessing creativity that is both automatic and objective, eliminating the need for human involvement in the scoring process. Even more importantly, linguistic analysis offers the possibility of being a fully valid form of creativity assessment, allowing for a new, more naturalistic assessment of human creativity.

## Data Availability Statement

The original contributions presented in the study are included in the article/supplementary material, further inquiries can be directed to the corresponding author.

## Author Contributions

GF supervised the design of the study, conducted additional analyses, and revised the manuscript. SA collected text samples, conducted analyses, and wrote up first draft of manuscript. Both authors contributed to the article and approved the submitted version.

## Conflict of Interest

The authors declare that the research was conducted in the absence of any commercial or financial relationships that could be construed as a potential conflict of interest.

## Publisher’s Note

All claims expressed in this article are solely those of the authors and do not necessarily represent those of their affiliated organizations, or those of the publisher, the editors and the reviewers. Any product that may be evaluated in this article, or claim that may be made by its manufacturer, is not guaranteed or endorsed by the publisher.

## Footnotes

1
https://www.nobelprize.org/
